# Open questions on the high-pressure chemistry of the noble gases

**DOI:** 10.1038/s42004-022-00631-5

**Published:** 2022-02-04

**Authors:** Maosheng Miao, Yuanhui Sun, Hanyu Liu, Yanming Ma

**Affiliations:** 1 Department of Chemistry and Biochemistry, California State University, Northridge, CA 91330 USA; 2 International Center for Computational Method & Software and State Key Laboratory of Superhard Materials, College of Physics, Jilin University, 130012 Changchun, China; 3 International Center of Future Science, Jilin University, 130012 Changchun, China

**Keywords:** Chemical bonding, Electronic properties and materials, Materials chemistry, Solid-state chemistry

## Abstract

Recent high-pressure studies have uncovered many types of chemical bonds present in noble gas compounds. Here, by extrapolating what has been found so far, the authors discuss which future discoveries can be expected and recommend further avenues of exploration.

Noble gas (NG) chemistry started from a theoretical prediction. Despite the lack of any known NG compound and the contemporaneously established atomic shell theory that supported the chemical inertness of NG elements, Linus Pauling predicted that F and O can oxidize Xe and Kr in 1933^1^, and he was proven right by Neil Bartlett who synthesized the first sets of NG compounds including XePtF_6_, XeF_2_, XeF_4_, etc, only 30 years later^2^. Since then, hundreds of NG compounds have been obtained, bulging out as a new branch of chemistry. However, in almost all these compounds, NG elements react as reductants and form bonds by donating or sharing their electrons, portraying them as “humble gases” instead of “noble gases” in the 100-year study of their chemistry. For comparison, almost all the other elements including the most active metals such as Cs can accommodate an electron and become Cs^−^^3^.

The recent high-pressure study of NG chemistry, which is again led by theoretical predictions, broke this standstill. Compared with the time of Pauling, we are now equipped with much more powerful tools, including first-principles density functional theory (DFT) calculations and various crystal structure search algorithms^4^. In around a decade, not only has this new approach led to the predictions and syntheses of numerous new compounds, but more importantly, it revealed many new types of NG bonds^5,6^. Almost all the bond types, including negatively charged NG, strong NG-NG covalent bonds, and H-bond-like NG bonds, can now be found in NG compounds under high pressure. The most striking of all is probably the revelation of an entirely new type of chemical binding, namely NG elements can form highly stable compounds without forming chemical bonds of any type.

## The range of oxidation states of NG elements

**Fig. 1:**
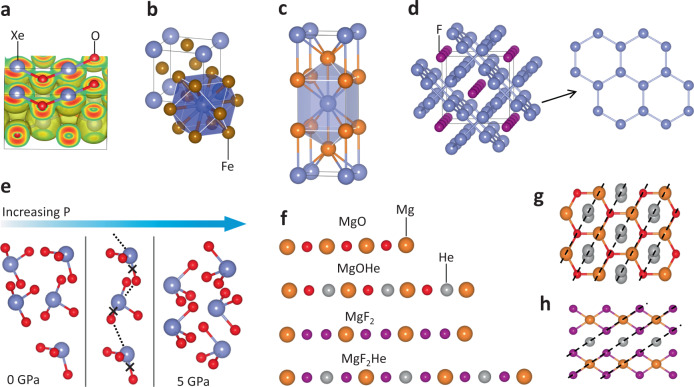
Stable structures with noble gas elements acting as cations, anions, and insertions. **a** Electron localization function of XeO in Pbcm structure, showing Xe-O chains connected by strong Xe-O covalent bonds. **b** $P\bar 62m$ structure of XeFe_3_, with Xe atom located at the center of XeFe_12_ tetrakaidecahedron. **c** I4/mmm structure of Mg_2_Xe. **d** I4/mcm structure of Xe_2_F, in which the Xe atoms form graphene-like Xe monolayers with strong Xe-Xe bonds. **e** The migration path of O via noble gas bond in the transition from low-pressure phase to high-pressure phase of XeO_3_. **f** Schematic diagram of different types of He insertion in AB-type (MgO) and AB_2_-type (MgF_2_) compounds. **g** (001) plane of MgOHe, the He dimers locate out of the Mg-O chains (black dash lines) to avoid the increasing of electrostatic Madelung energy. **h** (110) plane of MgF_2_He, the He atoms locate between two F atoms on the F-Mg-F chains (black dashed lines) to reduce the electrostatic Madelung energy.

The extension of chemistry is largely defined by the achievable oxidation states of the elements. Pressure has been shown to stabilize NG elements in various oxidations states in compounds such as oxides (Fig. 1a)^7–9^ and fluorides^10^. Furthermore, NG elements might be oxidized by weaker oxidants, such as N^11^, C^12^, or even 3*d* metals^13^. It has been predicted that Xe can form stable compounds with Fe and Ni at the conditions of the Earth’s core (Fig. 1b)^13^, providing compelling evidence for the existence of chemical reservoirs that account for missing Xe in the core^14^. The ultimate open question remains what are the achievable oxidation states of all the NG elements and how do they depend on oxidants and on pressure. The reason that many NG elements can be oxidized to a higher oxidation state under pressure is because their outmost shell orbitals have a core that increases in energy more significantly than O and F 2*p* orbitals under pressure^15^. However, this general enhancement of oxidation strength under pressure has a limit and is counterbalanced by the polymerization of oxidant elements, such as O and F^6^. It is indeed not clear whether light NG elements such as Ne can be oxidized at all, no matter how high the pressure is.

NG elements also show another trend under high pressure, namely that they can gain electrons and become negatively charged while they react with active metals, such as Li and Mg^16–18^. For example, Mg and several NG elements including Xe, Kr, and Ar were predicted to form stable MgNG and Mg_2_NG compounds (Fig. 1c) under pressures higher than 125, 250, and 250 GPa, respectively^16^. The charge transfer from Mg to Xe is comparable to that from Mg to O in MgO at ambient pressure. In contrast to oxidation, these reduction reactions of NG elements have not been thoroughly explored and there is still a lack of experimental confirmation. Again, the major open question is the limit of the negative charges on NG elements and their relationship to external pressure.

## The allowable bonds in NG compounds

Many chemical bonds may form with zero or very little charge transfer, such as homonuclear bonds, hydrogen bonds, etc. NG-NG bonds can form only while their electrons are partially depleted from the shell^19^. The first example of this kind under pressure was found for the Xe-Xe bond in Xe_2_F that becomes stable at 60 GPa, in which Xe forms graphitic layers bonded by strong Xe-Xe bonds (Fig. 1d)^10^. The enhanced stability of the Xe-Xe bond is a major reason that XeF_2_, an archetype Xe-F compound, becomes unstable and is predicted to decompose into Xe_2_F and XeF_4_ at 81 GPa, which causes an experimentally observed insulator–metal transition^20^. However, the formation of Xe_2_F and the corresponding crystal structure have not yet been confirmed by high-pressure experiments. Naturally, the open question for the next step is whether other NG-NG bonds can be found in a stable compound, including both homonuclear and heteronuclear ones.

Another bond type that has also been missing in NG compounds is the type that is similar to hydrogen bonds. Indeed, such non-covalent bonds have been found for almost all families of elements and dubbed as halogen bonds, chalcogen bonds, pnictogen bonds, etc. Recent studies showed that the interactions between XeO_3_ molecules in XeO_3_ molecular crystals possess all the features of hydrogen bonds^21^. Especially, under increasing pressure, intramolecular Xe-O bonds are elongated, and the corresponding vibrational modes are softened^22^. This strong NG bond also provides a transition path of O during the phase transition from the low-pressure to the high-pressure structure of XeO_3_, which is similar to hydrogen bond-assisted proton transfers (Fig. 1e). There is no reason that such strong non-covalent bond types should be limited to only Xe-O. They might be even stronger for lighter NG elements such as Kr and Ar. But so far, none have been explored by DFT calculations or high-pressure experiments.

The most striking phenomenon in NG chemistry is probably a chemical binding force that can stabilize NG compounds without forming any local chemical bond^23^. Among all NG elements, He and Ne are the chemically most inert ones, and no stable solid compound of them was known, except that they can be inserted into some compounds with large voids. It was quite a surprise that Na and He were found to form stable compounds under high pressure with a substantial driving force^24^. At almost the same time, He was found to react with H_2_O and some binary ionic compounds, forming stable ternary compounds^25,26^. The mechanism of these NG insertion reactions was found to be the reduction of the long-range electrostatic energy (Madelung energy) while inserting He and Ne into the crystal lattice of ionic compounds with unequal numbers of cations and anions (Fig. 1f–h)^23^. Under the same mechanism, He was found to react with FeO_2_ that was recently proposed as an important mineral in the Earth’s lower mantle, rendering it a large chemical reservoir of He^27^. Most of the theoretical predictions have not been demonstrated by experiments, except the reaction with Na^24^. Again, the range of potential NG insertion is unclear, and we lack an estimation of which NG elements can be inserted, although so far only compounds with He and Ne insertions are known. Some states formed by He and H_2_O under pressure were found to be superionic^28^, but the extension of such states at high pressure and high temperature in various He-inserted compounds is still to be explored.

## Applications of NG chemistry under pressure

It is commonly believed that a novel compound synthesized under high-pressure conditions can only be useful if it remains stable or at least metastable after the pressure is released. This seems very discouraging to the application of high-pressure NG chemistry since most of the novel bonds can only form under high pressure. Strikingly, this trend of NG compounds can be utilized to obtain novel compounds or novel structures of a compound that cannot be directly attained under ambient conditions. For example, while inserting He, we may obtain a novel polynitrogen structure under pressure^29^. Although the compound is not stable and will lose He after releasing pressure, the remaining polynitrogen solid remains metastable, which provides a unique route to obtain new high-energy density materials. Similar methods could be applied to semiconductor materials such as Si, which could lead to new structures with desired properties such as a very different energy gap^30^. This is an unexplored direction and the potential of achieving novel structures of many traditional semiconductors, such as GaS, GaN, ZnO TiO_2_, etc. has not yet been studied. Again, DFT-based crystal structure predictions can lead the research but should be followed by experimental demonstrations.

NG elements are important geoscience markers and their distribution and abundance provide an important record of the formation and evolution of the Earth. An understanding of this record demands knowledge of the chemical affinity of the elements and their compounds under the high-pressure conditions of the Earth’s interior. The recent discovery that Xe and He can react with Fe, FeO_2_, and FeO_2_H under the conditions of the Earth’s interior showed that the core and the lower mantle of the Earth can be a chemical reservoir of these NG elements^13,27,31,32^, which offers an insightful explanation of the observed NG distribution. This research is far from being complete since the chemical reactivity of NG elements with various minerals such as MgSiO_3_ and Fe cores containing light elements under high pressure have not been explored yet.

## Outlook

In the foreseeable future, high-pressure studies will continuously extend the scope of NG chemistry by discovering new compounds with atypical compositions, a plethora of novel polyatomic species, and unusual oxidation states for these elements. While most of the past research has focused on the reactivity of NGs with a single element, there is a strong need to study the reactivity of NG with compounds, especially functional and mineral materials. This is a very challenging task even for DFT simulations since they often need to deal with the structure predictions of more complicated ternary and quaternary compounds. The understanding of the changes of bonds, the chemical characteristics of elements, the volume effects, and the interplay of all these factors under pressure can help to greatly reduce the effort of blindfolded structure search and therefore has become ever important. Moreover, the temperature is also another critical factor to change the reactivity of NG elements. Given that the Earth’s interior is under high temperature and high-pressure conditions, more studies are needed for NG chemistry under elevated temperatures and pressures.

## References

[1] Pauling L (1933). The formulas of antimonic acid and the antimonates. J. Am. Chem. Soc..

[2] Bartlett, N. Xenon hexafluoroplatinate (V) Xe^+^PtF_6_. In *Proc. Chemical Society* 218 (Chemical Society, 1962).

[3] Ellaboudy A, Dye JL, Smith PB (1983). Cesium 18-crown-6 compounds. A crystalline ceside and a crystalline electride. J. Am. Chem. Soc..

[4] Zhang, L., Wang, Y., Lv, J. & Ma, Y. Materials discovery at high pressures. *Nat. Rev. Mater.* **2**, 17005 (2017).

[5] Miao, M. Noble gases in solid compounds show a rich display of chemistry with enough pressure. *Front. Chem*. **8**, 570492 (2020). 10.3389/fchem.2020.570492 PMC7674853 33251181

[6] Miao M, Sun Y, Zurek E, Lin H (2020). Chemistry under high pressure. Nat. Rev. Chem..

[7] Zhu Q (2013). Stability of xenon oxides at high pressures. Nat. Chem..

[8] Dewaele A (2016). Synthesis and stability of xenon oxides Xe_2_O_5_ and Xe_3_O_2_ under pressure. Nat. Chem..

[9] Zaleski-Ejgierd P, Lata PM (2016). Krypton oxides under pressure. Sci. Rep..

[10] Peng F, Botana J, Wang Y, Ma Y, Miao M (2016). Unexpected trend in stability of Xe-F compounds under pressure driven by Xe-Xe covalent bonds. J. Phys. Chem. Lett..

[11] Peng, F., Wang, Y. C., Wang, H., Zhang, Y. W. & Ma, Y. M. Stable xenon nitride at high pressures. *Phys. Rev. B* **92**, 094104 (2015).

[12] Bovornratanaraks T, Tsuppayakorn-aek P, Luo W, Ahuja R (2019). Ground–state structure of semiconducting and superconducting phases in xenon carbides at high pressure. Sci. Rep..

[13] Zhu L, Liu H, Pickard CJ, Zou G, Ma Y (2014). Reactions of xenon with iron and nickel are predicted in the Earth’s inner core. Nat. Chem..

[14] Jephcoat AP (1998). Rare-gas solids in the Earth’s deep interior. Nature.

[15] Miao M (2013). Caesium in high oxidation states and as a *p*-block element. Nat. Chem..

[16] Miao MS (2015). Anionic chemistry of noble gases: formation of Mg-NG (NG = Xe, Kr, Ar) compounds under pressure. J. Am. Chem. Soc..

[17] Li X (2015). Stable lithium argon compounds under high pressure. Sci. Rep..

[18] Liu, Z., Botana, J., Miao, M. S. & Yan, D. D. Unexpected Xe anions in XeLin intermetallic compounds. *EPL* **117**, 26002 (2017).

[19] Drews T, Seppelt K (1997). The Xe ion—preparation and structure. Angew. Chem. Int. Ed..

[20] Kim M, Debessai M, Yoo CS (2010). Two- and three-dimensional extended solids and metallization of compressed XeF_2_. Nat. Chem..

[21] Bauzá A, Frontera A (2015). Aerogen bonding interaction: a new supramolecular force?. Angew. Chem. Int. Ed..

[22] Hou C, Wang X, Botana J, Miao M (2017). Noble gas bond and the behaviour of XeO_3_ under pressure. Phys. Chem. Chem. Phys..

[23] Liu Z (2018). Reactivity of He with ionic compounds under high pressure. Nat. Commun..

[24] Dong X (2017). A stable compound of helium and sodium at high pressure. Nat. Chem..

[25] Liu, H. Y., Yao, Y. S. & Klug, D. D. Stable structures of He and H_2_O at high pressure. *Phys. Rev. B* **91**, 014102 (2015).

[26] Gao H, Sun J, Pickard CJ, Needs RJ (2019). Prediction of pressure-induced stabilization of noble-gas-atom compounds with alkali oxides and alkali sulfides. Phys. Rev. Mater..

[27] Zhang J (2018). Rare helium-bearing compound FeO_2_He stabilized at Deep-Earth conditions. Phys. Rev. Lett..

[28] Liu, C. et al. Multiple superionic states in helium–water compounds. *Nat. Phys.*10.1038/s41567-019-0568-7 (2019).

[29] Li Y (2018). Route to high-energy density polymeric nitrogen t-N via He-N compounds. Nat. Commun..

[30] Bi, Y., Xu, E., Strobel, T. A. & Li, T. Formation of inclusion type silicon phases induced by inert gases. *Commun. Chem.* **1**, 15 (2018).

[31] Peng F (2020). Xenon iron oxides predicted as potential Xe hosts in Earth’s lower mantle. Nat. Commun..

[32] Zhang, J., Liu, H., Ma, Y. & Chen, C. Direct H-He chemical association in superionic FeO_2_H_2_He at Deep-Earth conditions. *Natl Sci. Rev.*10.1093/nsr/nwab168 (2021). 10.1093/nsr/nwab168 PMC9344844 35928982

